# Supertetrahedral polyanionic network in the first lithium phosphidoindate Li_3_InP_2_ – structural similarity to Li_2_SiP_2_ and Li_2_GeP_2_ and dissimilarity to Li_3_AlP_2_ and Li_3_GaP_2_†

**DOI:** 10.1039/d0sc05851c

**Published:** 2020-11-27

**Authors:** Tassilo M. F. Restle, Volker L. Deringer, Jan Meyer, Gabriele Raudaschl-Sieber, Thomas F. Fässler

**Affiliations:** Department of Chemistry, Technische Universität München Lichtenbergstraße 4 D-85747 Garching Germany Thomas.Faessler@lrz.tum.de; Department of Chemistry, University of Oxford South Parks Road Oxford OX1 3QR UK

## Abstract

Phosphide-based materials have been investigated as promising candidates for solid electrolytes, among which the recently reported Li_9_AlP_4_ displays an ionic conductivity of 3 mS cm^−1^. While the phases Li–Al–P and Li–Ga–P have already been investigated, no ternary indium-based phosphide has been reported up to now. Here, we describe the synthesis and characterization of the first lithium phosphidoindate Li_3_InP_2_, which is easily accessible *via* ball milling of the elements and subsequent annealing. Li_3_InP_2_ crystallizes in the tetragonal space group *I*4_1_/*acd* with lattice parameters of *a* = 12.0007(2) and *c* = 23.917(5) Å, featuring a supertetrahedral polyanionic framework of interconnected InP_4_ tetrahedra. All lithium atoms occupy tetrahedral voids with no partial occupation. Remarkably, Li_3_InP_2_ is not isotypic to the previously reported homologues Li_3_AlP_2_ and Li_3_GaP_2_, which both crystallize in the space group *Cmce* and feature 2D layers of connected tetrahedra but no supertetrahedral framework. DFT computations support the observed stability of Li_3_InP_2_. A detailed geometrical analysis leads to a more general insight into the structural factors governing lithium ion mobility in phosphide-based materials: in the non-ionic conducting Li_3_InP_2_ the Li ions exclusively occupy tetrahedral voids in the distorted close packing of P atoms, whereas partially filled octahedral voids are present in the moderate ionic conductors Li_2_SiP_2_ and Li_2_GeP_2_.

## Abstract 2

Li_3_InP_2_ exhibits a polyanionic framework of corner-sharing InP_4_ tetrahedra and DFT computations reveal the stability trend for indium in the tetragonal structure compared to the orthorhombic structure of the lighter homologues.

## Graphical abstract

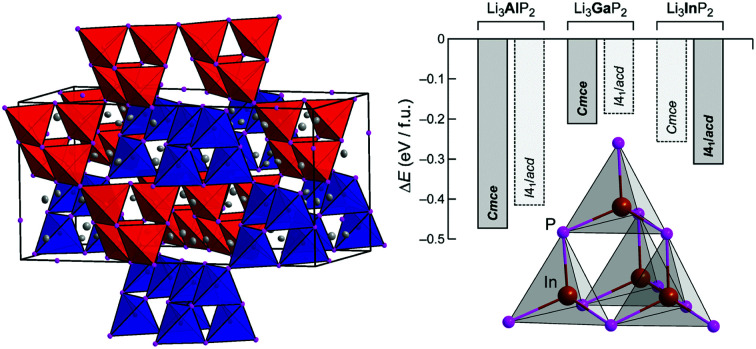

## Introduction

All-solid-state batteries (ASSB) have recently become the focus of research as an attractive alternative to state-of-the-art liquid-based batteries due to their enhanced safety combined with high energy/power density and mechanical stability.^1–7^ One of the main obstacles for the commercialization of ASSBs is the difficulty to develop superionic solid conductors, which are crucial for fast ionic diffusion in ASSBs. Recently, our group investigated new classes of lithium ion conductors based on phosphides. Starting with Li_8_SiP_4_ in 2016, we introduced phosphidosilicates with an ionic conductivity of 4.5 × 10^−5^ S cm^−1^.^8^ Lately, in the Li-richer compound Li_14_SiP_6_ the conductivity was even higher with up to 1 × 10^−3^ S cm^−1^.^9^ Furthermore, we extended the system to the heavier tetrel (group-14) homologues, phosphidogermanates, with two Li-ion conducting modifications of Li_8_GeP_4_ that show ionic conductivities of up to 8.6 × 10^−5^ S cm^−1^ and with Li_14_GeP_6_, which achieves an ionic conductivity of 1.7 × 10^−3^ S cm^−1^.^10,11^ The structural building units in these phosphides are [TtP_4_]^8−^ tetrahedra surrounded by lithium atoms (Tt = Si, Ge). They exhibit a huge structural variety, and by decreasing the amount of lithium, condensed and covalently connected tetrahedra are formed, thereby offering different polyanionic networks: Li_10_Si_2_P_6_ features pairs of edge-sharing SiP_4_ tetrahedra,^12^ in Li_2_SiP_2_/Li_2_GeP_2_ and LiSi_2_P_3_, respectively, SiP_4_ and GeP_4_ tetrahedra are condensed to networks of supertetrahedra.^8,13,14^ Layered structures have been reported as well: in Li_3_Si_3_P_7_, vertex-sharing SiP_4_ tetrahedra form double layers,^12^ and LiGe_3_P_3_ is built up by a two dimensionally extended polyanion comprising GeP_4_ and Ge(P_3_Ge) tetrahedra.^13^

Phosphide-based materials as lithium ionic conductors originated from the aliovalent substitution of [TtS_4_]^4−^ tetrahedra, which are the main building block in sulfide-based conductors. This leads to analogous structures with more negatively charged [TtP_4_]^8−^ tetrahedra, which can therefore accommodate more lithium than the well-known sulfur-based analogues. In recent investigations we expanded this class of compound further to phosphidoaluminates, which contain tetrahedral AlP_4_ building units, and we discovered the fast lithium ion conductor Li_9_AlP_4_, which shows ionic conductivities of 3 × 10^−3^ S cm^−1^.^15^ In addition, we also obtained Li_3_AlP_2_, which is built up by 
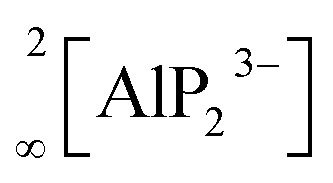
 layers of corner- and edge-sharing AlP_4_ tetrahedra,^16^ and we then also introduced the isotypic gallium compound Li_3_GaP_2_ as the first phosphidogallate.^16^ Both trielate (Tr = Al, Ga) compounds do not show moderate lithium ion conductivity but unexpectedly turned out to be direct band gap semiconductors with optical band gaps of 3.1 and 2.8 eV, respectively.

Prior to the present work, no ternary Li–In–P phase has been described in the literature, and only one ternary Na–In–P phase was mentioned: Na_3_InP_2_ is built up by a distorted hcp of P atoms with all octahedral voids filled by Na, whereas the tetrahedral voids are occupied by Na and In, giving rise to a polyanionic network of corner-sharing InP_4_ tetrahedra.^17^

In the present work, we report the first lithium phosphidoindate, Li_3_InP_2_, synthesized *via* ball milling of the elements and subsequent annealing. The compound retains the principal structural building unit of TrP_4_ tetrahedra, but their arrangement is notably different from that of Li_3_AlP_2_ and Li_3_GaP_2_. In Li_3_InP_2_, the InP_4_ tetrahedra are condensed to supertetrahedra in a three-dimensional framework. The structure is determined by single crystal X-ray diffraction and analyzed by complementary solid-state NMR experiments and first-principles computations. The knowledge of the lithium ion mobilities of the now-completed series of phosphidotrielates allows us to suggest a structural design rule linked to ionic conductivity, namely, the presence (or absence) of partially occupied Li-containing octahedral sites between which the ions can move rapidly.

## Results and discussion

### Synthesis and structure of Li_3_InP_2_

Li_3_InP_2_ was synthesized from the elements *via* a two-step procedure. At first, stoichiometric amounts of lithium, indium and phosphorus were ball milled resulting in a reactive mixture. Besides small amounts of the desired phase, Li_3_InP_2_, the polycrystalline powder contains considerable amounts of InP and Li_0.3_In_1.7_ (see Fig. S4†). Subsequently, pellets of the mixture were annealed in sealed niobium ampules at 1023 K for 22 h. Afterwards, the ampoules were rapidly cooled to room temperature by quenching in an ice-water mixture yielding almost phase-pure Li_3_InP_2_ with 3.3(1) % Li_0.3_In_1.7_ as an impurity according to Rietveld analysis (Fig. S3†). Annealing at lower temperatures such as 673 K or slow cooling rates led to impurities such as InP. Powdered Li_3_InP_2_ is brick-red. Complete data of the Rietveld refinement are given in the ESI; Tables S5 and S6.†

Red single crystals of Li_3_InP_2_ were obtained after reacting the elements with the formal stoichiometry “Li_3_In_2_P_3_” at 1073 K in tantalum ampoules. Besides Li_3_InP_2_, the resulting product contains InP and at least one more, so far unknown phase according to unassigned reflections in the powder X-ray diffractogram (see Fig. S5†). Details of the structure refinement of the single crystal X-ray diffraction data of Li_3_InP_2_ are listed in the ESI in Tables S1–S4.†

**Fig. 1:**
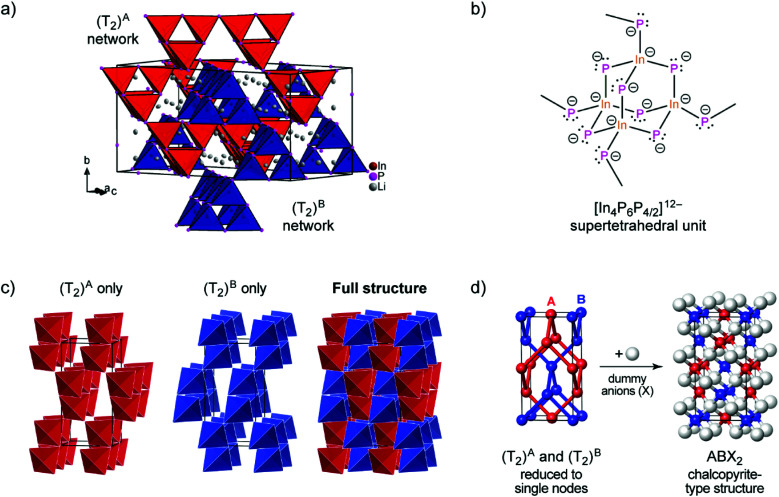
(a) The crystal structure of Li_3_InP_2_. The T2-supertetrahedra consist of four InP_4_ tetrahedra and form two independent adamantane-like networks (the two networks, denoted as (T_2_)^A^ and (T_2_)^B^, are shown in red and blue color, respectively). Li^+^ ions are located in tetrahedral voids of the distorted ccp of P atoms. Li, In and P atoms are depicted in grey, brown and purple color, respectively (displacement ellipsoids set at 90% at 150 K). Crystal data and structure refinement are shown in Tables S1–S4 in the ESI.† CSD 2026514 contains the ESI crystallographic data for this paper.† (b) Lewis structure with formal charges of the atoms. (c) The two independent adamantane-like supertetrahedral networks (T_2_)^A^ and (T_2_)^B^ and the penetration of the two networks (each T_2_ unit is represented by a tetrahedron). (d) A simplified view of the structure, in which the center of gravity of each T_2_ unit is represented by a colored sphere (“node”), inspired by ref. 18 Notice: the ABX_2_ chalcopyrite structure type is formed by the formal insertion of X atoms shown as grey spheres. Structural drawings in panels (c and d) were created using VESTA.^19^

According to the single crystal structure determination, Li_3_InP_2_ crystallizes in the tetragonal space group *I*4_1_/*acd* (no. 142) with seven independent crystallographic positions (one for In, three each for Li and P; Table S2†). Considering that the crystal structure is based on a tetragonally distorted cubic close packing of phosphorus atoms, the multiplicity of the phosphorus Wyckoff positions (32g + 16e + 16e) leads to a total of 128 tetrahedral voids and 64 octahedral voids. One quarter of these tetrahedral voids is filled by the indium atoms (Wyckoff position 32g). The remaining 96 tetrahedral voids are occupied by lithium (3 × 32g). Hence, the tetrahedral voids are fully occupied, whereas all octahedral voids are empty. The unit cell determined by single crystal X-ray diffraction is displayed in Fig. 1a.

Indium and phosphorus form InP_4_ tetrahedra, and four corner-sharing InP_4_ tetrahedra build a T2-supertetrahedron. These T2-supertetrahedra are interconnected *via* corners, yielding two independent adamantane-like networks, which are shown in red and blue colors in Fig. 1a and c.

The In and P atoms are covalently connected to four and two atoms, respectively, resulting in a formal negative charge for both In and P of (−1). Since the P atoms at the corner of the supertetrahedron are shared with the next supertetrahedron, one such unit can be written as [In_4_P_6_P_4/2_]^12−^ (Fig. 1b), which leads to an electronically balanced formula Li_3_InP_2_ (≡(Li^+^)_12_[In_4_P_6_P_4/2_]^12−^ or Li_12_In_4_P_8_).

The InP_4_ units slightly deviate from an ideal tetrahedron with P–In–P angles ranging from 107.20(1) to 111.55(1)°. The bond lengths within the InP_4_ tetrahedra are in the narrow range between 2.5676(5) and 2.5899(5) Å and are very similar to those in compounds with strong In–P interactions like InP (2.5412(1) Å)^20^ and Na_3_InP_2_ (2.592(3)–2.682(3) Å)^17^ and in excellent agreement with DFT computations after full structural optimization (2.57–2.58 Å). The Li–P bonds in Li_3_InP_2_ range from 2.526(2) to 2.673(2) Å and are in good agreement with those in other binary or ternary phases containing Li and P.^8–10,12,13,15^ DFT optimization yields 2.51–2.67 Å, again practically superimposable with the experimental results.

Considering each center of gravity of the supertetrahedra, the arrangement of the independent networks of the T2-supertetrahedra corresponds in a hierarchical relationship to the arrangement of the Cu and Fe cations in the chalcopyrite structure, which is highlighted in Fig. 1d. The concept of supertetrahedra is already known in the literature, including supertetrahedral sulfides,^21,22^ which show structures with huge cavities, and also phosphidosilicates.^8,23^

### MAS-NMR spectroscopy

**Fig. 2:**
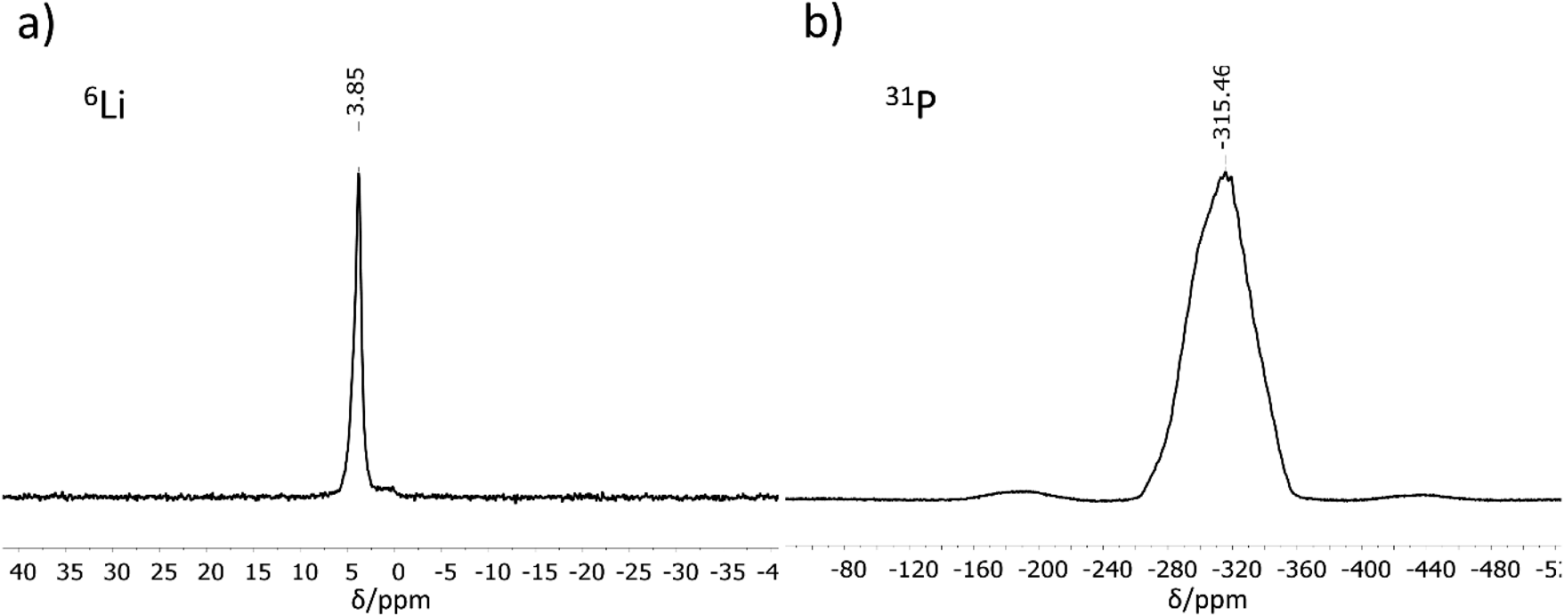
^6^Li (a) and ^31^P (b) MAS-NMR measurements performed for Li_3_InP_2_.

^6^Li and ^31^P MAS-NMR measurements (Fig. 2) support the results of the crystal structure determination. The ^6^Li NMR spectrum shows only one signal with a chemical shift of 3.85 ppm. As expected, the NMR experiment cannot distinguish between the three crystallographically different lithium atoms, all of which are tetrahedrally coordinated by phosphorus in a very similar chemical environment. The chemical shift of the Li atoms is in the same range as those for related compounds like Li_9_AlP_4_ (4.2 ppm), Li_3_AlP_2_ (4.0 and 3.0 ppm), Li_3_GaP_2_ (4.1 and 3.4 ppm), Li_2_SiP_2_ (2.1 ppm from ^7^Li MAS-NMR spectroscopy), and Li_2_GeP_2_ (3.6 and 2.4 ppm).^8,13,15,16^ Compared to the above-mentioned compounds with two signals in the ^6^Li NMR spectrum, the difference in local coordination, which is expressed by the P–Li–P angles, is the lowest for Li_3_InP_2_ (Li_3_InP_2_: 104.99(8)–113.25(8)°, Li_3_AlP_2_: 100.0(3)–116.647(1)°, Li_3_GaP_2_: 102.258(1)–115.2(3)°, Li_2_GeP_2_: 84.68(1)–158.89(2)°). The ^31^P NMR spectrum displays a very broad, asymmetric signal in the range of −260 to −360 ppm. This range is typical for chemical shifts of two-fold connected P^1−^ atoms such as in Li_3_AlP_2_ (−300 and −308.7 ppm) or Li_3_GaP_2_ (−234.8 and −280.5 ppm).^16^ However, the signals of two-fold connected P^1−^ atoms in the related phosphidotetrelates are much more downfield shifted (Li_2_SiP_2_: −129.1 and −241.5 ppm and Li_2_GeP_2_: −59.9, −164.8 and −178.4 ppm) due to the deshielding of the more electronegative tetrel elements compared to indium.^8,13^

Interestingly, only one ^31^P NMR signal is observed for Li_3_InP_2_, whereas two signals are obtained for all other related compounds. This correlates with the fact that the smallest distortion of the E–P–E bond angles is observed for Li_3_InP_2_ [106.411(9)–111.41(1)°] if compared to the others with E = Al, Ga, In, Si, Ge such as Li_3_AlP_2_ [78.298(1)–111.709(1)°], Li_3_GaP_2_ [79.943(1)–110.253(1)°], Li_2_SiP_2_ [102.669(9)–114.937(9)°], and Li_2_GeP_2_ [101.726(7)–112.609(7)°].

### Comparison of Li_3_InP_2_ with the lighter homologues Li_3_AlP_2_ and Li_3_GaP_2_

**Fig. 3:**
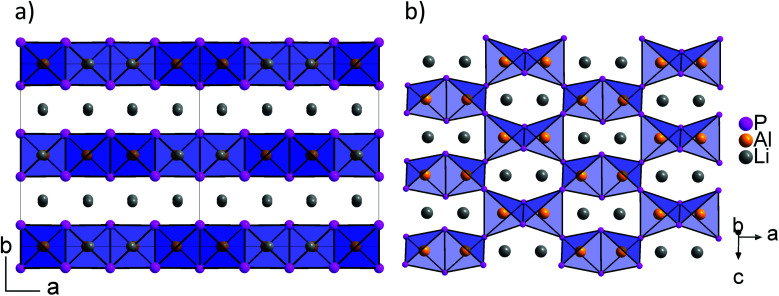
Structural details of Li_3_AlP_2_: (a) layers of 
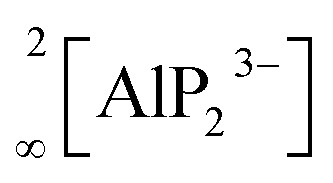
 polyanions are separated by lithium ions. (b) Top view of one 
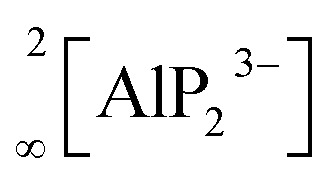
 layer of dimers of two edge-sharing AlP_4_ tetrahedra that are interconnected by vertex-sharing. Unfilled voids within the layer are filled with additional Li ions. AlP_4_ tetrahedra are shown as blue polyhedra. Al, P and Li atoms are drawn in orange, purple and grey, respectively.

Recently, we described the two isotypic phases Li_3_AlP_2_ and Li_3_GaP_2_,^16^ which crystallize in a distorted orthorhombic packing of phosphorus atoms in the space group *Cmce* (no. 64) with lattice parameters *a* = 11.5138(2), *b* = 11.7634(2), *c* = 5.8202(1) Å and *a* = 11.5839(2), *b* = 11.7809(2), *c* = 5.8129(2) Å, respectively, both determined by Rietveld refinement at room temperature. The crystal structures are built up by corner- and edge-sharing TrP_4_ (Tr = Al, Ga) tetrahedra in two-dimensional 
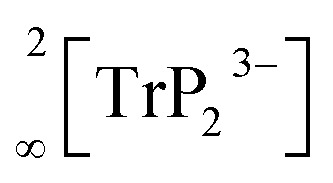
 layers. Based on a close packing of P atoms, the lithium atoms are located in all tetrahedral voids (Fig. 3). By contrast, Li_3_InP_2_ crystallizes in a tetragonal distorted phosphorus lattice in the space group *I*4_1_/*acd* (no. 142) with lattice parameters of *a* = 12.03049(8) and *c* = 23.9641(3) Å, determined by Rietveld refinement at room temperature, and as mentioned above, the single crystal structure determination reveals a three-dimensional structure with exclusively corner-sharing InP_4_ tetrahedra for t-Li_3_InP_2_ (Fig. 1).

**Fig. 4:**
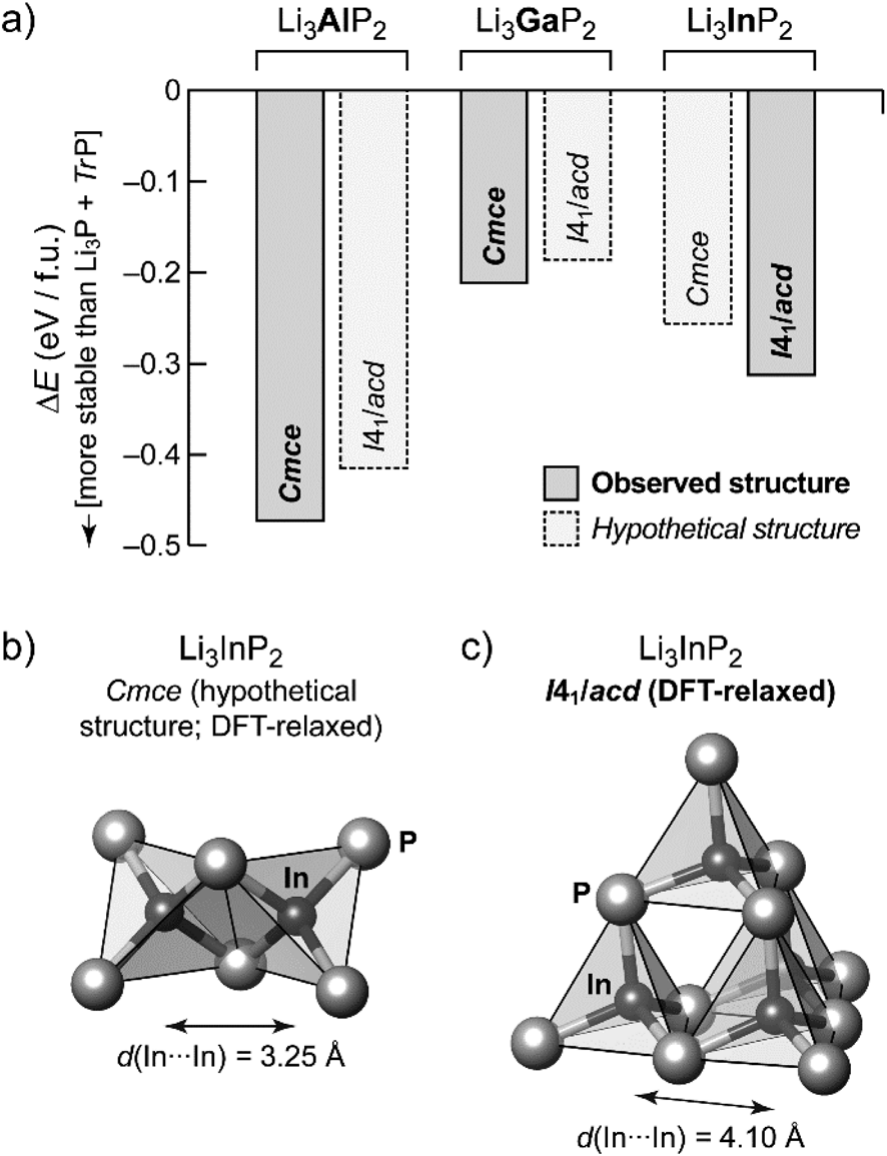
First-principles DFT computations for Li_3_AlP_2_, Li_3_GaP_2_ and Li_3_InP_2_. (a) Computed total energies for DFT-relaxed structural models of the Li_3_TrP_2_ (Tr = Al, Ga, or In). Results are given relative to the respective binary phases; negative values indicate that the formation of the ternary compound is favorable. Results for experimentally observed structures correspond to solid lines and darker shading. Hypothetical structures, generated by cation substitution and DFT relaxation, are shown by dashed lines and lighter shading, and in all three cases these are less favorable than their respective counterpart, in agreement with experimental observations. (b) Structural fragment from a hypothetical, DFT-generated, *Cmce* Li_3_InP_2_ structure, emphasizing the edge-sharing [InP_4_] tetrahedra, which may be compared to (c) the DFT-optimized *I*4_1_/*acd* Li_3_InP_2_ structure, from which a supertetrahedral fragment is shown.

In order to gain additional insight into the experimentally observed structure types, we performed DFT-based structural optimizations for the Al, Ga and In compounds using the PBEsol functional^24^ as implemented in CASTEP^25^ (computational details are given in the ESI†). In addition to the experimentally determined unit cells we performed a substitutional “cross-check”: both modifications, orthorhombic o-Li_3_TrP_2_ and tetragonal t-Li_3_TrP_2_, were used for Tr = Al, Ga and In, starting either from the experimentally determined structure or from a hypothetical one obtained by substituting the Tr species. The DFT-optimized cell parameters are in excellent agreement with the experiment for the title compound (we obtained *a*_DFT_ = 11.96 Å and *c*_DFT_ = 23.74 Å); full results are listed in Table S7.†Fig. 4a shows the resulting energies, relative to the respective binary phosphides similar in spirit to our recent work on Li_9_AlP_4_.^15^ We compute the DFT electronic energy, *E*, for the relaxed ternary structure as well as for Li_3_P and the respective zinc blende-type phase of AlP, GaP or InP; the difference (in the sense of a “reaction energy”) then allows us to estimate the stability of the ternary phase:Δ*E* = *E*(Li_3_TrP_2_) − [*E*(Li_3_P) + *E*(TrP)]

Negative values of Δ*E* therefore indicate that the ternary phase is stable with respect to the binaries (Fig. 4a).

The compounds Li_3_TrP_2_ are energetically favored over their respective binary components Li_3_P and AlP, GaP and InP. The latter all adapt the cubic zinc blende type. The energy gain is significant considering the known stability of the zinc blende type that is most frequent among III–V semiconductors. More importantly, the *difference* in pairs of Δ*E* values allows us to compare the tendency for assuming either the *Cmce* or the *I*4_1_/*acd* structure for all of the Li_3_TrP_2_ phases. For the Al and Ga compounds, the *Cmce* structure is favored by about 0.06 and 0.03 eV per formula unit (f.u.), respectively; by contrast, the *I*4_1_/*acd* structure is preferred for Li_3_InP_2_ (by about 0.06 eV f.u.^−1^), all in agreement with experiments. The stabilization of the title compound compared to the constituent binary phosphides is computed to be 0.31 eV f.u.^−1^ (indicated by a negative sign in the convention of Fig. 4a), which represents a significant gain in stability and explains the synthetic accessibility of the ternary compound. Whilst there will always remain a certain error due to the DFT approximation and the neglect of thermal effects, we do trust that the computed trends shown in Fig. 4a are robust, and we note that they are fully consistent with the experimental observations.

As expected, the unit cell volume for the heavier homologues increases, however the In compound shows a much stronger increase: 788.30 Å^3^ for Al and 793.28 Å^3^ for Ga if compared to 867.10 Å^3^ (=3468.39 Å^3^:4) for In. This correlates with a larger increase of the size of the InP_4_ tetrahedron (8.8857 Å^3^) compared to AlP_4_ (7.0897 Å^3^) and GaP_4_ (7.1334 Å^3^).

The trends of the interatomic Tr–Tr (Tr = Al, Ga, In) distances in Li_3_AlP_2_, Li_3_GaP_2_ and Li_3_InP_2_ are listed in Table 1. Regarding the different orthorhombic (Li_3_AlP_2_, Li_3_GaP_2_) and tetragonal structures (Li_3_InP_2_), the interatomic distances of the metal atoms are shorter in the orthorhombic structures, where edge-sharing tetrahedra occur compared to the tetragonal structure, where only corner-sharing tetrahedra are present. One may ask for the origin of the preference of one structure type over the other when comparing all three phosphidotrielates side-by-side. Interestingly, the results of the calculations are in agreement with Pauling's third rule. At least qualitatively and within the limits of such empirical concepts,^26^ edge-sharing tetrahedra are disfavored on account of the repulsion of positively charged central atoms (Fig. 4b and c). This effect might be expected to be strongest in the In compound, where not only the ionic radius is the largest of the three, but the computed Mulliken charges for the series of *Cmce* structures (Al: +0.42*e*, Ga: +0.57*e*, hypothetical In structure: +0.65*e*) appear to be consistent with an increasing repulsion of Tr atoms in the case of edge-sharing tetrahedra. Note that the Mulliken charges, derived from quantum-mechanical computation, are not to the same as the formal negative charge of the Tr atom using the Lewis valence model (Fig. 1b). Accordingly, a structure containing edge-sharing tetrahedra is observed for Li_3_AlP_2_ and Li_3_GaP_2_, but not for Li_3_InP_2_ (Fig. 4c). This trend of the differences of the different metal to metal distances by DFT calculation is confirmed by the experimental interatomic Tr–Tr (Tr = Al, Ga, In) distances (Table 1). The experimental In–In distance is significantly longer than the Al–Al or Ga–Ga distances (4.116(3) Å (In) *vs.* 3.028(5) Å (Al) and 3.089(2) Å (Ga)).

**Table 1:** Comparison of the shortest interatomic Tr–Tr (Tr = Al, Ga, In) distances, the cell volume and the volume per formula unit in Li_3_AlP_2_, Li_3_GaP_2_ and Li_3_InP_2_ from Rietveld refinements at room temperature^16^

	o-Li_3_AlP_2_	o-Li_3_GaP_2_	t-Li_3_InP_2_
Cell volume/Å^3^	788.29(2)	793.28(2)	3468.39(6) (3468.39:4 = 867.10)
Volume per formula unit/Å^3^	98.54	99.16	108.38
Tetrahedron volume/Å^3^	7.0897	7.1334	8.8857
Tr–Tr distances/Å	3.028(5)	3.089(2)	4.116(3)

### Comparison of Li_3_InP_2_ with the phosphidotetrelates Li_2_SiP_2_ and Li_2_GeP_2_

The crystal structure of Li_3_InP_2_ is related to the structure of Li_2_SiP_2_ and Li_2_GeP_2_.^8,13^ The two latter isotypic phases also crystallize in the space group *I*4_1_/*acd* (no. 142), with lattice parameters of *a* = 12.1111(1) and *c* = 18.6299(4) Å for Li_2_SiP_2_ and *a* = 12.3070(1) and *c* = 19.0307(4) Å for Li_2_GeP_2_ and a slightly longer *a*, but much shorter *c* parameter as compared to Li_3_InP_2_. A full comparison of the lattice parameters and the tetrahedral volumes in Li_3_InP_2_, Li_2_SiP_2_ and Li_2_GeP_2_ is given in Table 2.

**Table 2:** Comparison of the tetrahedral volumes of InP_4_, SiP_4_ and GeP_4_, the distances and angles between the supertetrahedra and the lithium coordination in Li_3_InP_2_, Li_2_SiP_2_ and Li_2_GeP_2_ obtained from single crystal structure determination. The values for Li_2_SiP_2_ and Li_2_GeP_2_ are taken from the literature^8,13^

	Li_3_InP_2_	Li_2_SiP_2_	Li_2_GeP_2_
*a*/Å	12.0007(2)	12.1111(1)	12.3070(1)
*c*/Å	23.917(5)	18.6299(4)	19.0307(4)
*V*/Å^3^	3447.7(1)	2732.61(7)	2882.42(9)
*V*_(Tr/Tt)P4 tetrahedra_/Å^3^	8.7944	5.8042	6.4083
Distances (Å) between the centers of the T2-supertetrahedra	8.4729(1), 8.4899(9)	7.6395(1), 8.5638(1)	7.7782(1), 8.7023(1)

Assuming an average volume of 18 Å^3^ per heavy atom, the increase in cell volume corresponds approximately to the volume of 32 additional lithium atoms in the unit cell of Li_2_SiP_2_. Besides the change in the number of Li atoms, also the larger volume of the InP_4_ tetrahedra compared to SiP_4_/GeP_4_ (see Table 2) contributes to an overall increase of the volume. However, this increase is highly anisotropic, since in Li_3_InP_2_ the lattice parameter *c* increases strongly, whereas the lattice parameter *a* is even slightly shorter compared to the one in Li_2_SiP_2_ and Li_2_GeP_2_.

**Fig. 5:**
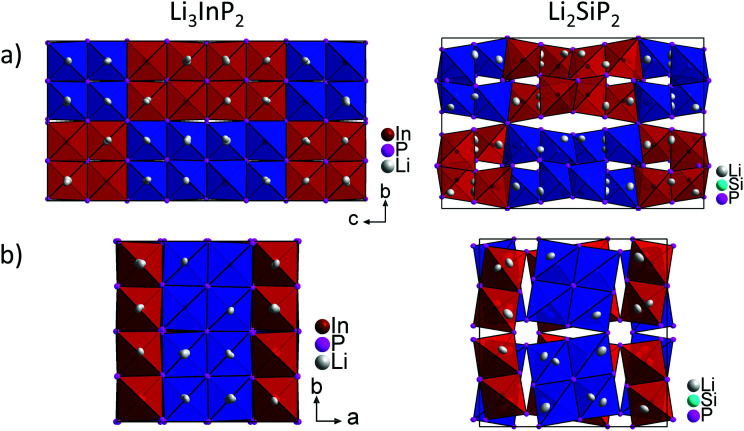
Comparison of the crystal structures of Li_3_InP_2_ (left) and Li_2_SiP_2_ (right). (a) The unit cell in *a* direction. (b) The unit cell in *c* direction. The InP_4_ and SiP_4_ tetrahedra are shown in blue and red. Both structures exhibit two independent diamond-like networks of T2-supertetrahedra. The different networks are drawn in blue and red, respectively.

Fig. 5 shows a comparison of the structures of Li_3_InP_2_ and Li_2_SiP_2_ viewed along the *a* and *c* direction. In Li_3_InP_2_ the InP_4_ tetrahedra respectively the T2-supertetrahedra are aligned in an almost parallel fashion, whereas in Li_2_SiP_2_ the T2-supertetrahedra are rotated along the tetragonal axes. Interestingly, the parallel alignment in Li_3_InP_2_ leads to a slight decrease of the *a* and *b* axes despite the higher lithium content, but to a significant increase of the *c* axes.

In Table 3 the Wyckoff positions in Li_3_InP_2_ and Li_2_SiP_2_ are compared (Li_2_GeP_2_ is omitted since it is isotypic to Li_2_SiP_2_). The higher Li content of the In compound arises from the occupation of two 32g Wyckoff sites instead of two 16f sites in the tetrelates. As a consequence, the coordination environments of the lithium atoms in the structures are different. The coordination of the lithium atoms in Li_3_InP_2_ and Li_2_SiP_2_ by phosphorus is illustrated in Fig. S2 and S8,† respectively. The positions Li1 and Li3 are similarly coordinated by four phosphorus atoms forming a distorted tetrahedron. By contrast, Li2 fills a strongly distorted octahedral void of phosphorus atoms with significant longer Li–P distances compared to Li1 and Li3. Here, the lithium atom Li2 is not located in the center of gravity of the octahedron but shows two much longer distances to neighboring P atoms of the distorted octahedron, resulting in a butterfly-type coordination of four P atoms. Interestingly, despite the smaller amount of Li atoms in Li_2_SiP_2_, not all the tetrahedral voids are occupied. In both compounds 25% of the tetrahedral voids are occupied by In or Si. Whereas all of the remaining 75% tetrahedral voids in Li_3_InP_2_ are filled with Li, only 37.5% are occupied by Li in Li_2_SiP_2_. In the latter, however, Li atoms occupy 25% of the distorted octahedral voids.

**Table 3:** Comparison of Wyckoff positions and atomic coordinates in Li_3_InP_2_ and Li_2_SiP_2_

Atom	Li_3_InP_2_				Li_2_SiP_2_			
	Wyck.	*x*	*y*	*z*	Wyck.	*x*	*y*	*z*
In1/Si	32g	0.11730(2)	0.12523(2)	0.31365(2)	32g	0.08345(2)	0.12967(2)	0.30710(1)
P1	16d	0	1/4	0.00065(2)	16d	0	1/4	0.23093(2)
P2	16e	0.23884(3)	0	1/4	16e	0.18335(3)	0	1/4
P3	32g	0.25031(2)	0.25604(2)	0.12516(2)	32g	0.21330(2)	0.21711(2)	0.37222(2)
Li1	**32g**	0.1279(2)	0.3720(2)	0.0615(1)	**16f**	0.0946(2)	0.3446(2)	1/8
Li2	**32g**	0.1179(2)	0.1250(2)	0.0651(1)	**16f**	0.1506(2)	0.0995(2)	1/8
Li3	32g	0.3635(2)	0.1212(2)	0.19053(9)	32g	0.3426(2)	0.1271(2)	0.2173(1)

**Fig. 6:**
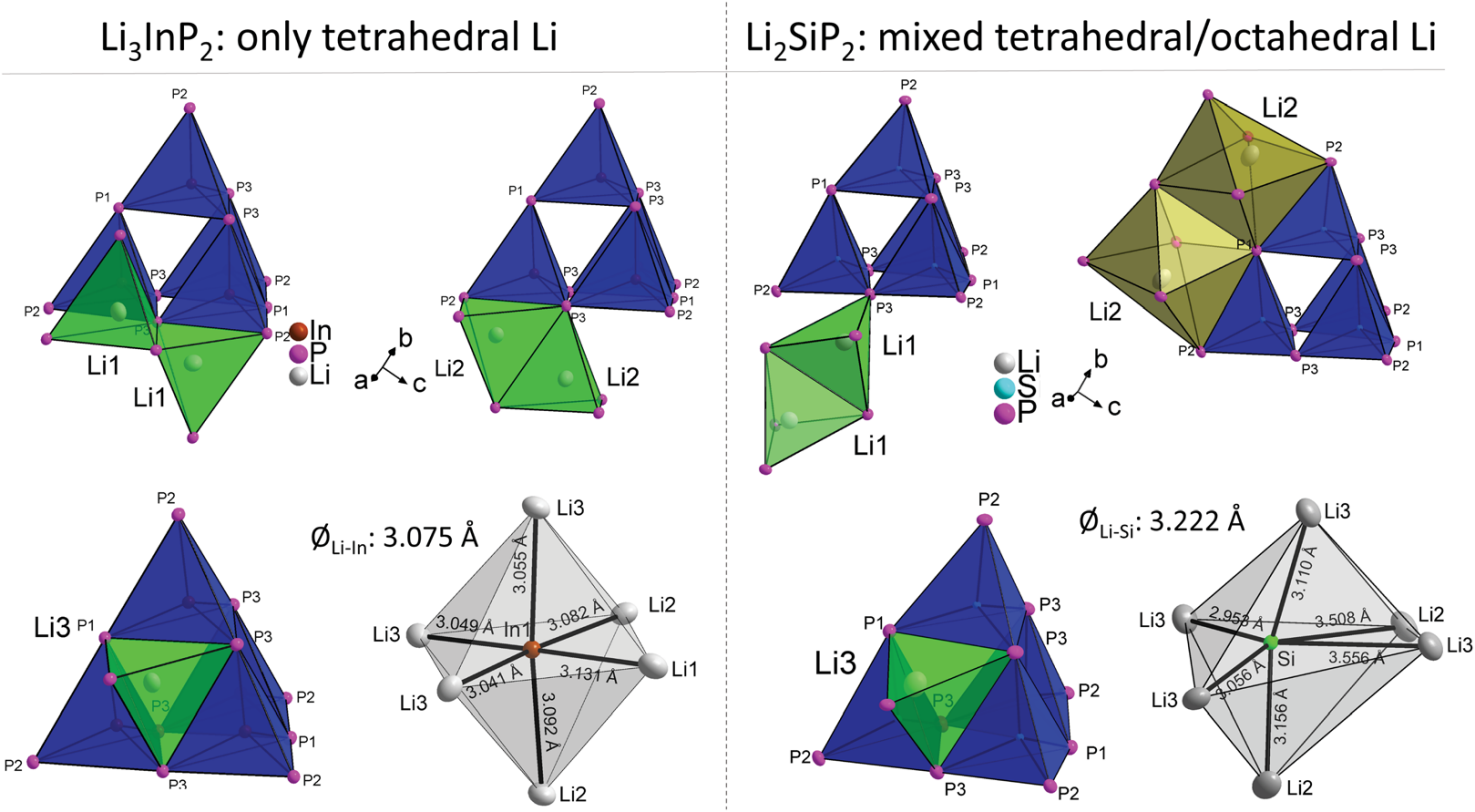
Comparison of the different lithium atoms and how they are connected to the T2-supertetrahedra in Li_3_InP_2_ (left) and Li_2_SiP_2_ (right). For clarity, just one or two polyhedra are shown for the lithium atoms, respectively. In Li_3_InP_2_, all lithium atoms (Li1, Li2 and Li3) are in tetrahedral voids. The tetrahedra of Li1 and Li2 are connected *via* one edge or one corner to the supertetrahedron. The tetrahedron of Li3 is connected *via* one face to the center of the supertetrahedron's face. In Li_2_SiP_2_, Li1 and Li3 are placed in tetrahedral voids (green) whereas Li2 resides in an octahedral void (orange). As in Li_3_InP_2_, the tetrahedron of Li3 is connected *via* one face to the center of the supertetrahedron's face. By contrast, the strongly distorted tetrahedron of Li1 is only connected *via* one corner to the supertetrahedron. The strongly distorted octahedra of Li2 are connected *via* edges to the supertetrahedron. In Li_3_InP_2_, the indium atoms are octahedrally coordinated by lithium atoms with an average Li–In distance of 3.075 Å. In Li_2_SiP_2_, the silicon atoms are coordinated by a strongly distorted octahedron of lithium atoms with an average Li–Si distance of 3.222 Å.

The different occupation of voids in Li_3_InP_2_ and Li_2_SiP_2_ also results in a different coordination of the supertetrahedra by lithium, which is shown in Fig. 6.

The different Li coordination arises from the different charges of the supertetrahedra Si_4_P_8_^8−^/Ge_4_P_8_^8−^ and In_4_P_8_^12−^ (Fig. 1b). In Li_3_InP_2_ the lithium atoms form an almost regular octahedron around the indium atom with In–Li distances in the narrow range of 3.041 to 3.131 Å with an average of 3.075 Å, whereas in Li_2_SiP_2_ the octahedron formed by lithium atoms around silicon is strongly distorted with longer average distances of 3.222 Å and values between 2.958 and 3.556 Å. As a consequence, also octahedral voids of P atoms are filled with Li ions in Li_2_SiP_2_.

### Impedance spectroscopy

For Li_3_InP_2_ two impedance measurements were performed to determine the ionic conductivity. The results are shown in Fig. S10.† The semi-circle can be described as parallel circuit element of a resistor and a constant phase element (*R*/*Q*). For the constant phase element the fit of the data acquired at 298 K resulted in *α* values of ≈0.99 and *Q* parameters of ≈2 × 10^−8^ F s^(*α* − 1)^; the conductivity was determined to *σ*(Li_3_InP_2_) = 2.8(2) × 10^−9^ S cm^−1^ at 298 K (obtained from two independently measured cells). DC polarization measurements in the range from 50 to 150 mV reveal an electronic conductivity of 2.7(3) × 10^−9^ S cm^−1^ at 298 K (based on the standard deviation of two cells). The conductivity value obtained by DC polarization measurements is in the same range as the value obtained by PEIS measurements. Hence, the Nyquist plot shows only the semi-circle of the electronic conductivity, and no semi-circle for the ionic conductivity appears.

## Conclusions

Li_3_InP_2_ is the first lithium phosphidoindate and can be described as a tetragonally distorted fcc lattice of P atoms (space group *I*4_1_/*acd*), in which the In atoms occupy tetrahedral voids, thus forming a polyanionic framework of InP_4_ supertetrahedra. The lithium atoms occupy the remaining tetrahedral voids. The structure of the compound is not isotypic to the previously reported ones of the lighter homologues, the orthorhombic compounds Li_3_AlP_2_ and Li_3_GaP_2_ (space group *Cmce*), which feature 2D layers of connected tetrahedra. First-principles DFT computations confirm the trend for the Al and Ga (In) compounds to crystallize in the orthorhombic (tetragonal) structure, respectively, which might originate in the different repulsive cation⋯cation interactions in both structures. Impedance spectroscopy reveals a very low electronic, but no ionic conductivity, whereas Li_2_SiP_2_ and Li_2_GeP_2_ show a moderate ionic mobility (2.2(3) × 10^−7^ S cm^−1^ at 293 K and 1.5(3) × 10^−7^ S cm^−1^ at 300 K, respectively).^8,13^ The geometrical analysis of the Li positions shows that in Li_3_InP_2_ all tetrahedral voids are fully occupied by lithium, whereas in Li_2_SiP_2_ and Li_2_GeP_2_ tetrahedral voids remain empty, and especially strongly distorted octahedral voids are filled. In accordance with the observations in fcc phosphide-based lithium ion conductors such as Li_9_AlP_4_,^15^ lithium diffusion preferably appears on pathways *via* partially occupied octahedral sites.

Overall, these results demonstrate that even though crystal structures of phosphide compounds can contain complex polyanionic networks, a relatively simple description in terms of distorted close-packed arrangements of phosphorus atoms gives better insight for the description of lithium ion mobility. The title compound Li_3_InP_2_ provides a missing link in two respects: (i) it shows the structure changes in the series Li_3_TrP_2_ for Tr = Al, Ga, In, and (ii) it shows changes in lithium ion mobility in the series Li_3_InP_2_, Li_2_SiP_2_ and Li_2_GeP_2_.

## Author contributions

TMFR carried out the crystal structure determination by single crystal and powder X-ray diffraction, performed the impedance spectroscopy measurements and wrote the manuscript draft. VLD carried out the DFT computations and provided discussion. JM contributed to the synthesis and data evaluation. GRS performed NMR experiments. TF designed research, provided guidance, and critically reviewed the manuscript.

## Conflicts of interest

The authors declare no competing financial interest.

## Supplementary Material

SC-012-D0SC05851C-s001

SC-012-D0SC05851C-s002
