# Supplementary material for: Supertetrahedral polyanionic network in the first lithium phosphidoindate Li3InP2 – structural similarity to Li2SiP2 and Li2GeP2 and dissimilarity to Li3AlP2 and Li3GaP2
Source: Chem Sci. 2020 Nov 27;12(4):1278–85. doi: 10.1039/d0sc05851c (PMC8179136; doi:10.1039/d0sc05851c)
Supplement: SC-012-D0SC05851C-s001 [file SC-012-D0SC05851C-s001.pdf]

Electronic Supplementary Material (ESI) for Chemical Science.
This journal is © The Royal Society of Chemistry 2020

ELECTRONIC SUPPLEMENTARY INFORMATION (ESI)

Supertetrahedral Polyanionic Network in the First Lithium Phosphidoindate Li_3InP_2 - Structural Similarity to Li_2SiP_2 and Li_2GeP_2 and Dissimilarity to Li_3AlP_2 and Li_3GaP_2

Tassilo M. F. Restle, Volker L. Deringer, Jan Meyer, Gabriele Raudaschl-Sieber, Thomas F. Fässler

---

Content: page

Experimental Section 1

Crystallographic Data 4

Results of the Rietveld Analysis 8

PXRD Measurements 10

DSC Measurement 11

DFT-optimized Lattice Parameters for Li_3AlP_2, Li_3GaP_2, Li_3InP_2 12

Structural Comparison to Li_2SiP_2 12

Impedance Spectroscopy 13

References 13

Experimental Section

All steps of synthesis and sample preparation were carried out inside an argon-filled glove box (MBraun, $p(H_2O)$, $p(O_2)$ < 0.1 ppm). Prior to use, lithium (Li, rods, Rockwood Lithium, > 99 %) was cleaned from oxide layers. Indium (In, drops, Sigma-Aldrich, 99.9 %), and phosphorus (P, powder, Sigma-Aldrich, 97 %) were used without any further purification.

Synthesis of Li_3InP_2. Li_3InP_2 was synthesized from the elements via ball milling and subsequent annealing, followed by rapid quenching. Lithium (318.6 mg, 45.4 mmol, 3.0 equiv.), indium (1741.0 mg, 15.2 mmol, 1.0 equiv.) and phosphorus (967.4 mg, 30.3 mmol, 2.0 equiv.) were ball milled (Fritsch Pulverisette 6) 72 times at 500 rpm with rest periods (every 10 min for 5 min) using a ZrO_2 milling set (45 mL jar and with 25 balls with a diameter of 1 cm). The obtained black-brown mixture was pressed into pellets with a diameter of 13 mm for 30 sec. at 5 t using a hydraulic press (Specac Atlas 15T). The fragmented pellets were filled into a niobium ampoule (8 mm diameter). The ampoule was sealed in an electric arc furnace (Edmund Bühler MAM1), enclosed in a quartz reaction container under vacuum and subsequently heated with 5 K·min^{-1} to 1023 K, and dwelled for 22 h in a tube furnace (HTM Reetz

Loba 1200-42-600-1-OW with a EUROTHERM S 14083 temperature controller). Afterwards, the ampoule was rapidly cooled by quenching in a water-ice mixture. After grinding the pellets, a brick-red powder was obtained. Li_3InP_2 is sensitive against moisture and air.

Red single crystals were obtained by the reaction of lithium, indium and phosphorus with the formal stoichiometry “$Li_3In_2P_3$” in a tantalum ampoule. Lithium (19.4 mg, 2.77 mmol, 3.0 equiv.), indium (211.5 mg, 1.84 mmol, 2.0 equiv.) and phosphorus (88.1 mg, 2.76 mmol, 3.0 equiv.) were filled into a tantalum ampoule. The ampoule was sealed in an electric arc furnace (Edmund Bühler MAM1), enclosed in a quartz reaction container under vacuum and subsequently heated with 5 $K\cdot min^{-1}$ to 1073 K, dwelled for 24 h and cooled with 0.5 $K\cdot min^{-1}$ to room temperature in a tube furnace (HTM Reetz Loba 1200-42-600-1-OW with a EUROTHERM S 14083 temperature controller), yielding a violet product with metallic lustre.

Powder X-ray diffraction. For powder X-ray diffraction (PXRD) measurements, the samples were ground in an agate mortar and sealed inside 0.3 mm glass capillaries. PXRD measurements were performed at room temperature on a STOE Stadi P diffractometer equipped with a Ge(111) monochromator for Mo $K_{\alpha 1}$ radiation (λ = 0.7093 Å) and a Dectris MYTHEN DCS 1K solid-state detector. The raw powder data were processed with the software package WinXPOW.[1]

Rietveld refinement. Rietveld refinements were performed with TOPAS V6.[2] The parameters of the single crystal structure determination were used as the structural model. All cell parameters and the atom positions were refined freely. Isotropic displacement parameters were refined independently for In, and for P and Li a joint displacement parameter was assigned.

Single crystal structure determination. A single crystal of Li_3InP_2 was sealed in a 0.3 mm glass capillary. The single crystal X-ray diffraction (SCXRD) measurement was performed on a STOE Stadivari diffractometer equipped with a Ge(111) monochromator, an Mo $K_{\alpha 1}$ radiation (λ = 0.71073 Å) source and a DECTRIS PILATUS3R 300 K detector. The structure was solved by Direct Methods (SHELXS) and refined by full-matrix least-squares calculations against F^2 (SHELXL).[3]

Differential scanning calorimetry (DSC). For thermal analysis, Li_3InP_2 was sealed in a niobium ampoule, and measured on a DSC machine (Netzsch, DSC 404 Pegasus) under a constant gas flow of 75 $mL\cdot min^{-1}$. The samples were heated to 1023 K and then cooled to 423 K twice at a rate of 5 $K\cdot min^{-1}$. To determine the onset temperatures of the DSC signals**,** the PROTEUS Thermal Analysis software was used.[4]

NMR spectroscopy. Magic angle spinning (MAS) NMR spectroscopy was carried out on a Bruker Avance 300 NMR device operating at 7.04 T by the use of a 4 mm ZrO_2 rotor. The resonance frequencies of the measured nuclei are 44.17 MHz and 121.46 MHz for ^6Li and ^{31}P, respectively. The rotational frequency was 15 kHz. The MAS spectra have been obtained at room temperature with relaxation delays of 10 s

(^{6}Li) and 30 s (^{31}P), and 880 scans (^{6}Li) and 360 scans (^{31}P). The spectra are referenced to LiCl (1 M, aq) and LiCl (s) offering chemical shifts of 0.0 ppm and -1.15 ppm, respectively. The ^{31}P spectrum is referred to ammonium dihydrogen phosphate (s) with a chemical shift of 1.11 ppm with reference to concentrated H_3PO_4. All spectra were recorded using single-pulse excitation.

Impedance spectroscopy. The electrochemical impedance spectroscopy for Li_3InP_2 was performed in an in-house designed cell. The detailed setup and procedure are described in Restle *et al.*.[5] Powdered samples of Li_3InP_2 (300 mg) were placed between two 8 mm dies, and the screws were fastened with a torque of 30 Nm, compressing the samples to 89 % of the theoretical density. Impedance spectra were recorded on a Bio-Logic potentiostat (SP-300) in a frequency range from 7 MHz to 50 mHz at a potentiostatic excitation of ±10 mV. Data were treated using the software EC-Lab (V 11.27). The measurements were performed in an Ar-filled glove box between 299 K. The electronic conductivity was determined with the same setup using a potentiostatic polarization procedure, applying voltages of 50, 100 and 150 mV for 7 h each.

Density-functional theory (DFT) computations. First-principles computations in the framework of DFT were carried out based on the experimentally obtained structural data and subsequent geometry optimization. The PBEsol functional[6] and on-the-fly ultrasoft pseudopotentials were employed as implemented in CASTEP.[7] Reciprocal space was sampled at the Γ point for structural relaxation of the title compound, on a 3×3×2 grid of points for a subsequent single-point energy evaluation, and on appropriately adjusted grids for all other compounds considered. The electronic cut-off energy for plane-wave expansions was 1000 eV, and all reported single-point energies are corrected for finite-basis-set effects.[8] Energies of formation were obtained relative to the elements (their modifications taken to be *bcc* Li, *fcc* Al, α-Ga, body-centered tetragonal In, and orthorhombic black P), and relative to the binary compounds (hexagonal Li_3P,[9] and zinc blende-type AlP, GaP, and InP). Mulliken charges on atoms were obtained as implemented in CASTEP.[10]

Crystallographic Data

Table S 1. Crystallographic data and refinement parameters of the SC-XRD analysis of Li_3InP_2.

Empirical formula	Li_3InP_2
Formula weight / g·mol^{-1}	197.58
Crystal size / mm^3	0.1 x 0.1 x 0.05
Crystal color	red
Crystal shape	block
T / K	150
Space group	$I4_1/acd$ (no. 142)
Unit cell dimension / Å	*a* = 12.0065(3)
	c = 23.9165(7)
V / Å^3	3447.7(2)
Z	32
ρ (calc.) / g·cm^{-3}	3.045
μ / mm^{-1}	5.993
Θ Range / deg	5.886 - 85.77
Index range *hkl*	-16 < *h* < 19
	-19 < *k* < 19
	-38 < *l* < 36
Reflections collected	67179
Independent reflections	39481
R_{int}	0.0331
Reflections with *I* > 2 *σ*(*I*)	1577
Data/restraints/parameter	1905/0/56
Absorption correction	Empirical
Goodness-of-fit on F^2	1.023
Final *R* indices [*I* > 2 *σ*(*I*)]^{a,b}	R_1 = 0.014
	wR_2 = 0.030
R indices (all data)^{a,b}	R_1 = 0.021
	wR_2 = 0.032
Largest diff. peak and hole/e·Å^{-3}	0.427/-0.818
Depository no.	CSD-2026514

$^a\, R_1 = \sum\left||F_o| - |F_c|\right| / \sum|F_o|$. $^b\, wR_2 = [\sum w(F_o^2 - F_c^2)^2 / \sum w(F_o^2)^2]^{1/2}$.

Table S 2. Atomic coordinates and isotropic displacement parameters for Li_3InP_2 from a single crystal structure determination at 150 K.

Atom	Wyckoff position	*x*	*y*	*z*	U_{iso}
In1	32g	0.11730(2)	0.12523(2)	0.31365(2)	0.00578(3)
P1	16d	0	¼	0.00065(2)	0.00610(8)
P2	16e	0.23884(3)	0	¼	0.00597(8)
P3	32g	0.25031(2)	0.25604(2)	0.12516(2)	0.00613(6)
Li1	32g	0.1279(2)	0.3720(2)	0.0615(1)	0.0143(5)
Li2	32g	0.1179(2)	0.1250(2)	0.0651(1)	0.0118(5)
Li3	32g	0.3635(2)	0.1212(2)	0.19053(9)	0.0111(4)

Table S 3. Anisotropic displacement parameters (Å^2) for Li_3InP_2 from a single crystal structure determination at 150 K.

Atom	U_{11}	U_{22}	U_{33}	U_{12}	U_{13}	U_{23}
In1	0.00608(4)	0.00592(4)	0.00532(4)	0.00011(4)	0.00006(3)	-0.00009(3)
P1	0.0066(2)	0.0062(2)	0.0054(2)	0.0008(1)	0.00000	0.00000
P2	0.0059(2)	0.00645(2)	0.0056(2)	0.00000	0.00000	-0.0007(1)
P3	0.0061(1)	0.0065(1)	0.0058(1)	-0.00002(9)	0.00000(9)	-0.00063(9)
Li1	0.013(1)	0.016(1)	0.014(1)	-0.001(1)	-0.004(1)	0.001(1)
Li2	0.009(1)	0.014(1)	0.012(1)	0.002(1)	-0.0003(8)	0.0000(9)
Li3	0.0127(9)	0.0093(9)	0.011(1)	-0.002(1)	-0.0014(8)	0.0019(9)

Table S 4. Selected interatomic distances up to 3 Å in Li_3InP_2 from a single crystal structure determination at 150 K.

atom pair			*d* / *Å*	atom pair			*d* / *Å*
In1	P1	1x	2.5676(3)	Li2	P3	1x	2.553(2)
	P3	1x	2.5704(3)		P1	1x	2.575(2)
	P3	1x	2.5885(3)		P2	1x	2.609(2)
	P2	1x	2.5898(2)		P3	1x	2.659(2)
	Li3	1x	3.041(2)		Li2	1x	2.868(6)
	Li3	1x	3.050(3)		Li3	1x	2.869(4)
	Li3	1x	3.055(2)		Li1	1x	2.953(3)
	Li2	1x	3.082(3)		Li1	1x	2.969(3)
	Li2	1x	3.092(2)		In1	1x	3.082(3)
	Li1	1x	3.131(2)		In1	1x	3.091(2)
P1	In1	2x	2.5676(3)	Li3	P2	1x	2.525(3)
	Li1	2x	2.573(2)		P3	1x	2.629(2)
	Li2	2x	2.575(2)		P3	1x	2.651(2)
	Li3	2x	2.674(2)		P1	1x	2.674(2)
P2	Li3	2x	2.525(3)		Li2	1x	2.869(4)
	In1	2x	2.5898(3)		Li1	1x	2.898(4)
	Li2	2x	2.609(2)		Li1	1x	2.926(3)
	Li1	2x	2.665(2)		In1	1x	3.041(2)
P3	Li1	1x	2.533(2)		In1	1x	3.050(3)
	Li2	1x	2.553(2)		In1	1x	3.055(2)
	In1	1x	2.5704(3)				
	In1	1x	2.5885(3)				
	Li1	1x	2.610(2)				
	Li3	1x	2.629(2)				
	Li3	1x	2.651(2)				
	Li2	1x	2.659(2)				
Li1	P3	1x	2.533(2)				
	P1	1x	2.573(2)				
	P3	1x	2.610(2)				
	P2	2x	2.665(2)				
	Li3	1x	2.898(4)				
	Li3	1x	2.926(3)				
	Li2	1x	2.953(3)				
	Li2	1x	2.969(3)				
	Li1	1x	3.039(4)				
	In1	1x	3.131(2)				

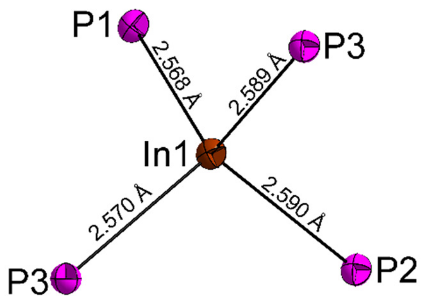

Figure S 1. Coordination polyhedron of the In atom by phosphorus in Li_3InP_2. The neighbor's phosphorus atoms form slightly distorted tetrahedra.

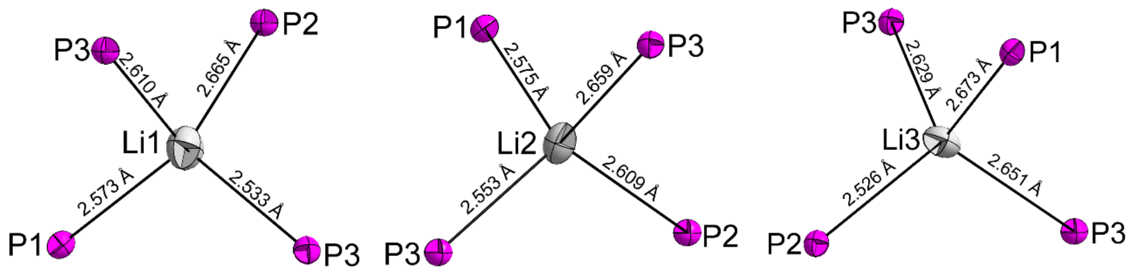

Figure S 2. Coordination polyhedra of the Li atoms by phosphorus in Li_3InP_2. The neighbor's phosphorus atoms form slightly distorted tetrahedra.

Rietveld Analysis

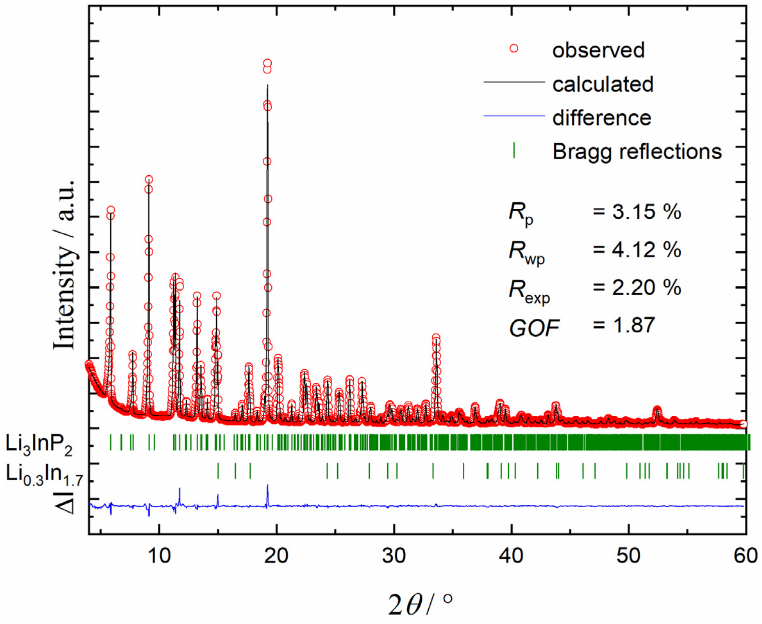

Figure S 3. Rietveld analysis of the powder X-ray diffractogram of Li_3InP_2. The red line indicates the observed intensities, the black line the calculated intensities and the blue line the difference between both. Bragg positions are marked by green dashes. The ratio of Li_3InP_2 to $Li_{0.3}In_{1.7}$ is 96.7(1) wt.-%: 3.3(1) wt.-%.

Table S 5. Crystallographic data of Li_3InP_2 obtained from powder XRD by Rietveld refinement.

Empirical formula	Li_3InP_2
Formula weight	197.58 g·mol^{-1}
Temperature	293 K
Color	red
Crystal system	tetragonal
Space group	$I41/acd$ (no. 142)
Lattice parameters	*a* = 12.03049(8) Å
	c = 23.9641(3) Å
V	3468.39(6) Å^3
Z	32
ρ (calc.)	3.02715(5) g·cm^{-3}
Wavelength	0.70930 Å
Θ Range	3.997 – 59.812 deg
R_p	0.0315
R_{wp}	0.0412
R_{exp}	0.0220
GOF	1.871

Table S 6. Atomic coordinates and isotropic displacement parameters for Li_3InP_2 from Rietveld analysis.

**Atom**	**Wyckoff position**	***x***	***y***	***z***	U_{iso}
In1	32g	0.1171(1)	0.1253(1)	0.31358(7)	0.0059(2)
P1	16d	0	¼	0.0012 (3)	0.0053(3)
P2	16e	0.2395(4)	0	¼	0.0053(3)
P3	32g	0.2501(3)	0.2567(3)	0.1249(4)	0.0053(3)
Li1	32g	0.132(2)	0.368(2)	0.060(2)	0.003(2)
Li2	32g	0.116(3)	0.122(2)	0.066(1)	0.003(2)
Li3	32g	0.370(2)	0.122(3)	0.193(1)	0.003(2)

PXRD Measurements

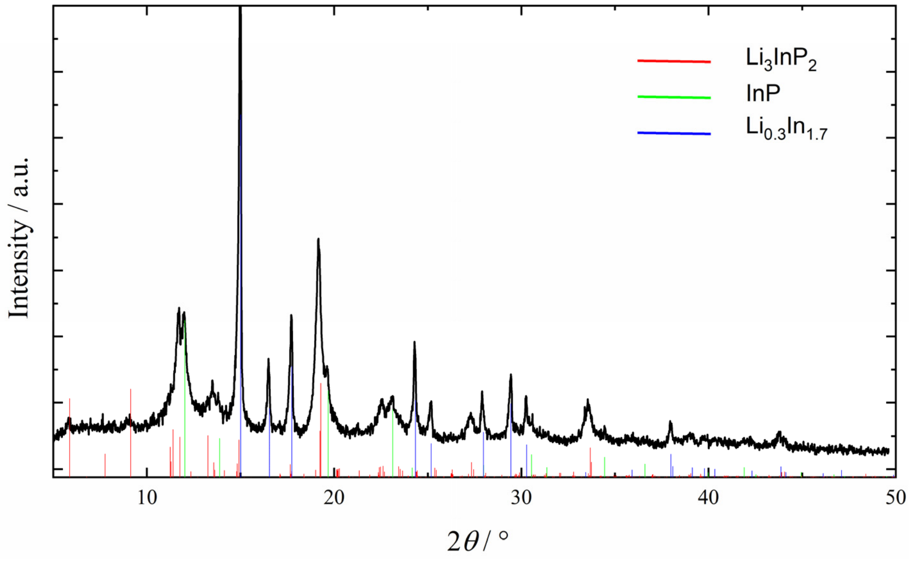

Figure S 4. Experimental powder X-ray diffraction pattern of the product of ball milled elements in a stochiometric ratio of 3 Li + In + 2 P.

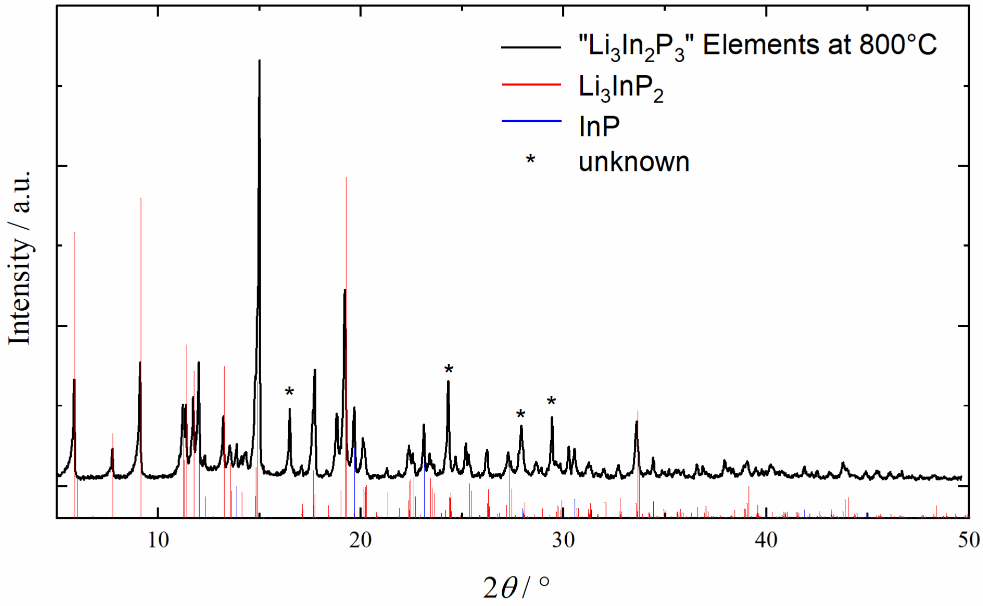

Figure S 5. Experimental powder X-ray diffraction pattern of the product of the reaction 3 Li + 2 In + 3 P at 1073 K in a tantalum ampoule.

DSC Measurement

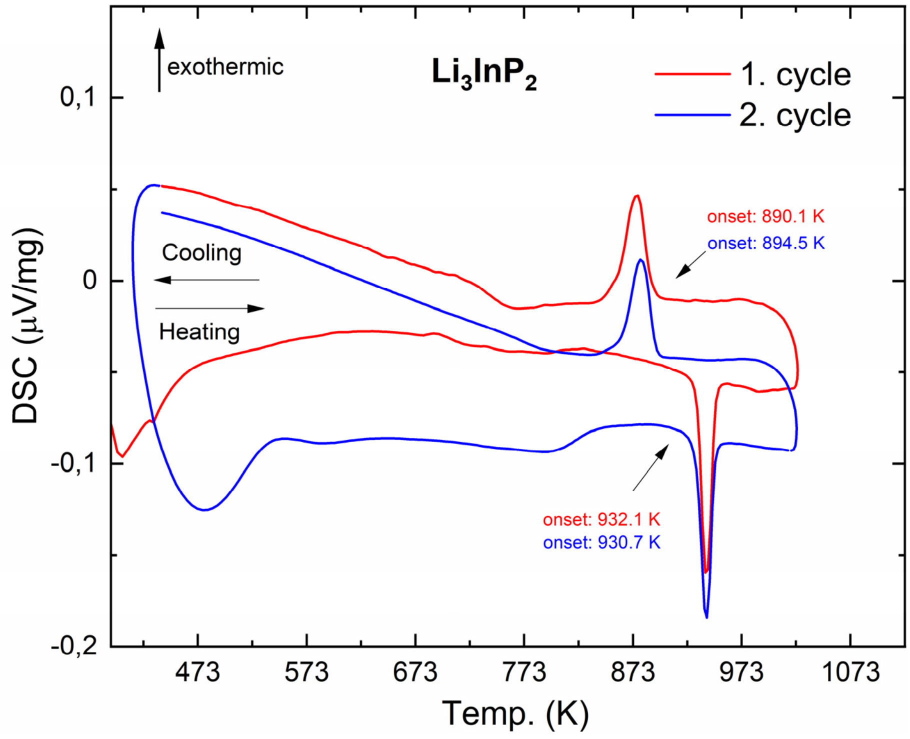

Figure S 6. DSC curves of the reactive mixture of Li_3InP_2. Two measurement cycles were performed using heating/cooling rates of 5 K·min^{-1}. The reversible signal at around 930 K might belong to the melting process of Li_3InP_2. The PXRD after the DSC measurement (Figure S7) looks equal to the PXRD before the measurement.

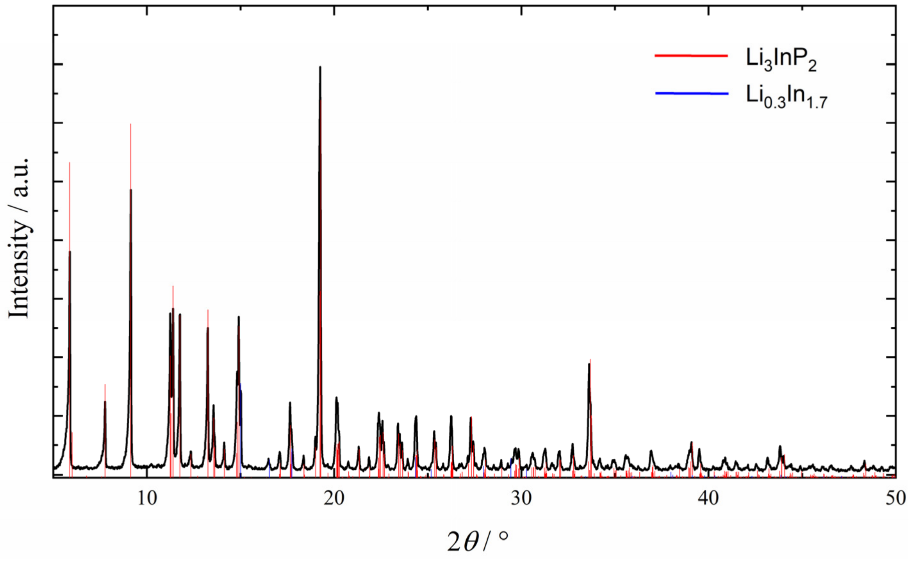

Figure S 7. Experimental powder X-ray diffraction pattern of the product of the DSC measurement.

DFT-optimized lattice parameters for Li_3AlP_2, Li_3GaP_2 and Li_3InP_2

Table S 7. DFT-optimized lattice parameters for Li_3AlP_2, Li_3GaP_2 and Li_3InP_2 in the space groups *Cmce* and *I*4$_1$*/acd*, respectively. Boldface entries emphasize the structure types, which were found by experiments. Italic entries emphasize the hypothetical structures obtained by substitution into homologous structures and subsequent relaxation.

	Cmce			*I*4$_1$*/acd*		
	o-Li_3AlP_2	*o*-Li_3GaP_2	*o*-Li_3InP_2	*t*-Li_3AlP_2	*t*-Li_3GaP_2	*t*-Li_3InP_2
a / Å	**11.439**	**11.517**	*11.991*	*11.564*	*11.584*	**11.959**
b / Å	**11.660**	**11.656**	*11.953*	*11.564*	*11.584*	**11.959**
c / Å	**5.772**	**5.763**	*5.968*	*22.835*	*22.833*	**23.743**
V / Å^3	**769.86**	**773.64**	*88.38*	*3053.64*	*3063.94*	**3395.67**

Structural Comparison to Li_2SiP_2

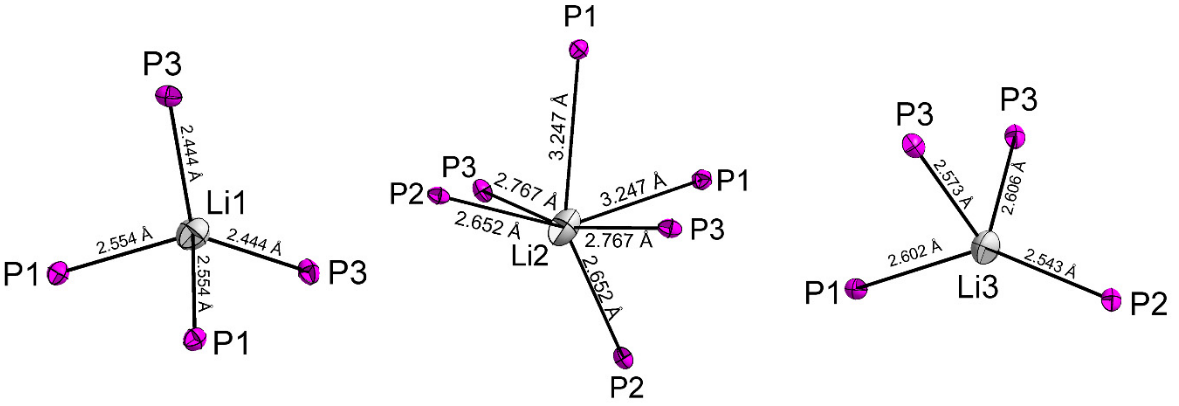

Figure S 8. Coordination polyhedra of the Li atoms by phosphorus in Li_2SiP_2. The neighbor's phosphorus atoms form strongly distorted tetrahedra for Li1 and Li2 and strongly distorted octahedra for Li2.

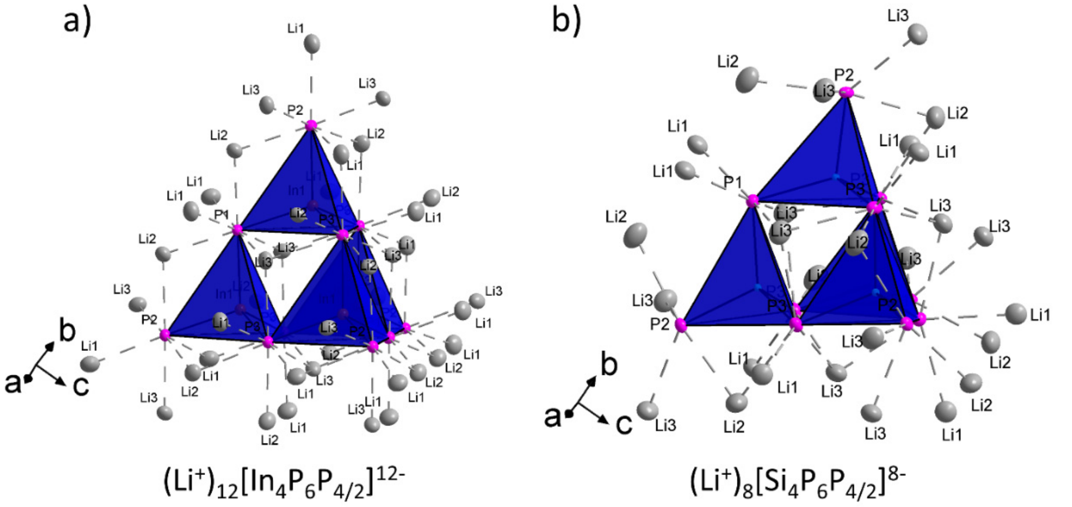

Figure S 9. Comparison of the lithium atoms in the first coordination sphere of the supertetrahedra in Li_3InP_2 (a) and Li_2SiP_2 (b).

Impedance Spectroscopy

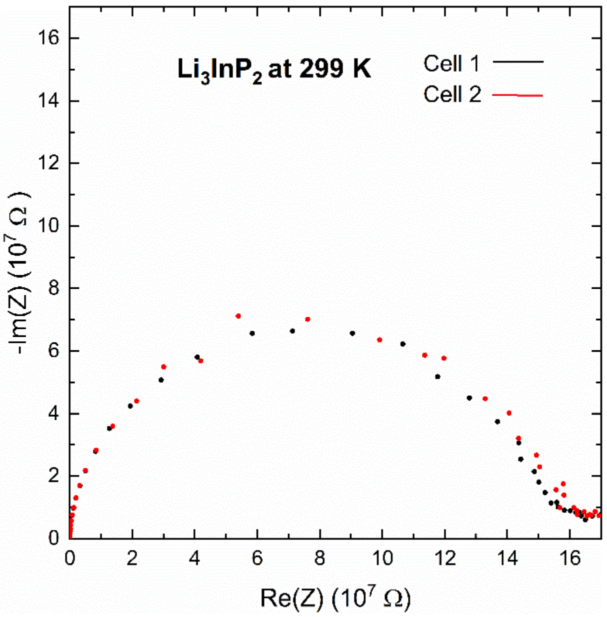

Figure S 10. Nyquist plot of Li_3InP_2 measured under blocking conditions and recorded at 299 K.

References

[1] *WinXPOW,* 3.0.2.1, STOE & Cie GmbH, Darmstadt, Germany, **2011**.

[2] *TOPAS,* 6, Bruker AXS, **2016**.

[3] G. Sheldrick, *Acta Crystallogr., Sect. C: Struct. Chem.* **2015**, *71*, 3-8.

[4] *PROTEUS Thermal Analysis V4.8.2,* Netzsch-Gerätebau GmbH, Selb, **2006**.

[5] T. M. F. Restle, C. Sedlmeier, H. Kirchhain, W. Klein, G. Raudaschl-Sieber, V. L. Deringer, L. v. Wüllen, H. A. Gasteiger, T. F. Fässler, *Angew. Chem. Int. Ed.* **2020**, *59*, 5665-5674.

[6] J. P. Perdew, A. Ruzsinszky, G. I. Csonka, O. A. Vydrov, G. E. Scuseria, L. A. Constantin, X. Zhou, K. Burke, *Phys. Rev. Lett.* **2008**, *100*, 136406.

[7] S. J. Clark, M. D. Segall, C. J. Pickard, P. J. Hasnip, M. J. Probert, K. Refson, M. C. Payne, *Z. Krist.* **2005**, *220*, 567-570.

[8] G. P. Francis, M. C. Payne, *J. Phys.-Condes. Matter* **1990**, *2*, 4395-4404.

[9] Y. Dong, F. J. DiSalvo, *Acta Crystallogr., Sect. E* **2007**, *63*, i97--i98.

[10] M. D. Segall, R. Shah, C. J. Pickard, M. C. Payne, *Phys. Rev. B* **1996**, *54*, 16317-16320.
